# Antimicrobial properties of *Fomitopsis officinalis* in the light of its bioactive metabolites: a review

**DOI:** 10.1080/21501203.2018.1536680

**Published:** 2018-10-25

**Authors:** Carolina Girometta

**Affiliations:** Department of Earth and Environmental Sciences, Università degli Studi di Pavia, Pavia, Italy

**Keywords:** *Fomitopsis officinalis*, *Laricifomes officinalis*, antimicrobial, antibacterial, antiviral, trypanocidal, coumarins, lanostane triterpenoids

## Abstract

*Fomitopsis officinalis*, also known as *Laricifomes officinalis*, is a medicinal polypore used for millennia (*Agarikon*) to contrast several diseases, particularly the pulmonary ones. A rich literature has dealt with its ethno-mycological aspects, but isolation and chemical characterisation of single compounds has only recently significantly developed, as well as *in vitro* tests for bioactivity. According to several reports there is evidence of a broad-spectrum antibacterial and antiviral activity by *F. officinalis*, including pathogens like *Mycobacterium tuberculosis, Yersinia pseudotuberculosis* and *Staphylococcus aureus*, as well as *Ortopox* virus. Chlorinated coumarins from mycelia and lanostane triterpenoids from basidiomes have been demonstrated to be directly responsible for antiviral-antibacterial and trypanocidal activity, respectively. A wider literature deals instead with crude extracts including an undetermined mixture of metabolites, whose efficacy *in vitro* is yet far from being standardised as extraction and treatment methodology are highly variable. Nevertheless, *in vivo* tests on bees provided promising results in order to develop sustainable solutions against the pathogens responsible for colony collapse disorders. Despite increasing attention has been paid to other medicinal aspects of this polypore, such as immune-tropic or antitumor, this review rationally reports and critically analyses the available knowledge by focusing on aspects of antimicrobial properties.

## 1. Introduction

Plants and fungi represent a major source of bioactive molecules showing antimicrobial properties. At least a part of such metabolites evolved as a response against a broad spectrum of microbial and nonmicrobial antagonists and this makes them particularly interesting also when dealing with pathogenic targets of human and animal concern (Wasser 2014; Mithöfer and Boland 2012; Gargano et al. 2017).

From a scientific point of view, species that have been reported as effective by traditional medicine when separately administered represent a more promising topic than species adopted in multi-ingredient formulates, where the single contributes are hardly distinguishable.

*Fomitopsis officinalis* (Batsch) (Bondartsev & Singer) is a polyporoid fungus recognised as medicinal for millennia all over its distribution range, particularly against pulmonary diseases. The first written report about the so-called Agarikon is by the protopharmacologist Dioscorides Pedanios (I century AD) but the use of this unmistakable species has thereafter been documented on Alps, Central and Eastern Europe, Urals, Siberia and North America, where it sometimes acquired mythic and ritual significance (Blanchette et al. 1992).

Far beyond the folk superstition, bioactive molecules have been recently isolated from this fungus and proved to show remarkable antimicrobial effect (Grienke et al. 2014; Stamets 2018).

This species is also known by the name of *Laricifomes officinalis* (Batsch) (Kotl. & Pouzar), that has been proposed to replace *F. officinalis* since molecular data suggest a separate systematic position from Fomitopsidaceae (Han et al. 2016b).

Showing a holartic distribution, *F. officinalis* is a wood-decay species developing its basidiomata on Pinaceae, where it causes heart cubic brown rot as a slow-growing necrotrophic parasite. Interestingly, several conifers are reported as host in North America, whereas in Eurasia *F. officinalis* almost exclusively occurs on *Larix* spp. Basidiomata are perennial and forward their growth for several years, sometimes reaching a considerable size. Apart from the morphological features of fully developed specimens, *F. officinalis* is immediately distinguished from other species by the chalky consistence, flavour and smell even in primordial stage (Bernicchia 2005; Ryvarden and Melo 2014).

Thus, due to its easy identification and consolidated use in folk medicine, *F. officinalis* has gained popularity both at international and local levels. Nevertheless, scientific literature dealing with chemical characterisation of metabolites and *in vitro* tests on biological properties has only recently developed.

Moreover, only a few purified compounds have been up to now singularly tested, that is the respective contribute of each compound to bioactivity has been poorly investigated.

Aim of the present review is to provide a rational and critical discussion on the available knowledge about the antimicrobial properties of *F. officinalis* in the light of the secondary metabolites characterisation.

## 2. Secondary metabolites in *Fomitopsis officinalis* showing antimicrobial activity

A complete review of secondary metabolites from *F. officinalis* and other medicinal polypores until 2013 was provided by (Grienke et al. 2014); an updated list is reported in Table 1. Although a plethora of molecules has been isolated and chemically characterised from the structural point of view, only a minority was tested as it concerns antimicrobial properties.

**Table 1.** Secondary metabolites isolated from *F. officinalis.*

Molecule	References	Antimicrobial properties
Eburicoic acid	Wu et al. 2005 and Shi et al. 2017	–
Sulfurenic acid	Wu et al. 2005	–
Fomefficinic acid A-E	Wu et al. 2004	–
Versisponic acid D	Wu et al. 2005	–
Fomefficinic acid F,G	Wu et al. 2009 and Naranmandakh et al. 2018	–
Dehydroeburicoic acid	Wu et al. 2005, Feng et al. 2010 and Shi et al. 2017	–
Dehydrosulfurenic acid	Wu et al. 2005 and Feng et al. 2010	–
3-Ketodehydrosulfurenic acid	Wu et al. 2005, Feng et al. 2010 and Shi et al. 2017	–
Fomefficinol A-B	Wu et al. 2009	–
Fomlactone A-C	Wu et al. 2009	–
Obtusifoldienol	Epstein and Van Lear 1966	–
Eburicodiol	Anderson and Epstein 1971	–
(3β,5α,6β)-3,6-dihydroxy-4,4,14-trimethyl-Pregn-8- en-20-one	Anderson et al. 1972	–
3α-hydroxy-4,4,14α-trimethyl-5α-pregn-8-en-20- one	Epstein and Van Lear 1966	–
Eburical	Anderson and Epstein 1971	–
Agaric acid	Airapetova et al. 2010 and Airapetova and Gromovykh. 2013	–
Laricinolic acid	Erb et al. 2000 and Wu et al. 2009	–
Officinalic acid	Epstein et al. (1979), Erb et al. (2000) and Wu et al. (2009)	–
2H-6-chloro-2-oxo-4-phenyl-1-Benzopyran-3- carboxylicacidethylester	Hwang et al. 2013	Antimicrobial
6-Chloro-4-phenyl-coumarin	Hwang et al. 2013	Antimicrobial
Officimalonic acids A-H	Han et al. 2016	–
Fomitopsin A	Han et al. 2016	–
Fomitopsin C	Shi et al. 2017	–
Fomitopsin F, G, H	Naranmandakh et al. 2018	Trypanocidal
Demalonyl fomitopsin H	Naranmandakh et al. 2018	Trypanocidal
Fomitopsin D Ethyl ester	Naranmandakh et al. 2018	Trypanocidal
3-acetyloxylanosta-8, 24-dien-21-oic acid	Shi et al. 2017	–
(25S)-(+)-12α-hydroxy-3α- malonyloxy-24-methyllanosta-8,24(31)-dien-26-oic acid	Naranmandakh et al. 2018	–
15α-hydroxy- 3-oxo-24-methylenelanosta-7,9(11)-dien-21-oic acid	Wu et al. 2004, Han et al. 2016 and Naranmandakh et al. 2018	Trypanocidal
t-Z-trans-zeatin	Vedenicheva et al. 2016	–
c-Z-cis-zeatin	Vedenicheva et al. 2016	–
ZR-zeatin riboside	Vedenicheva et al. 2016	–
zeatin-О-glucoside	Vedenicheva et al. 2016	–

### 2.1. Chlorinated coumarins

**Figure 1.**
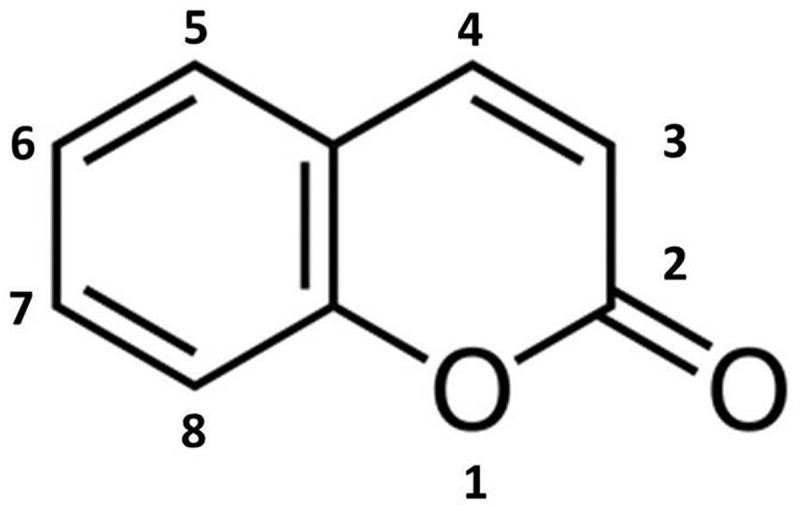
Simple coumarin or benzopyrone.

Two new chlorinated coumarins have been isolated from mycelia of *F. officinalis* by (Hwang et al. 2013) resembling a substituted derivative of simple coumarin (Figure 1).

In their simplest form coumarins are heterocyclic compounds basically constituted by a benzene ring and a pyrone one (benzopyrone). A wide range of derivatives including furan rings and pyran rings has been reported from bacteria, fungi and plants. As it regards fungi, coumarins have been up to now isolated from several species both in Ascomycota and Basidiomycota, including polypores (Costa et al. 2016); nevertheless, chlorination has been reported from *F. officinalis* only, despite chlorinated coumarins are known in plants (Guz et al. 2001; Patnam et al. 2005).

The biological activity of coumarins has been referred to a wide range of pharmacological properties, including antiviral, antibacterial, antifungal, antiparasitic and antielmintic. Chlorination and bromination (halogen substituents on an aromatic ring) are generally regarded as factors increasing antimicrobial activity as a whole (Basanagouda et al. 2009; Asif 2015; Al-Majedy et al. 2017).

**Figure 2.**
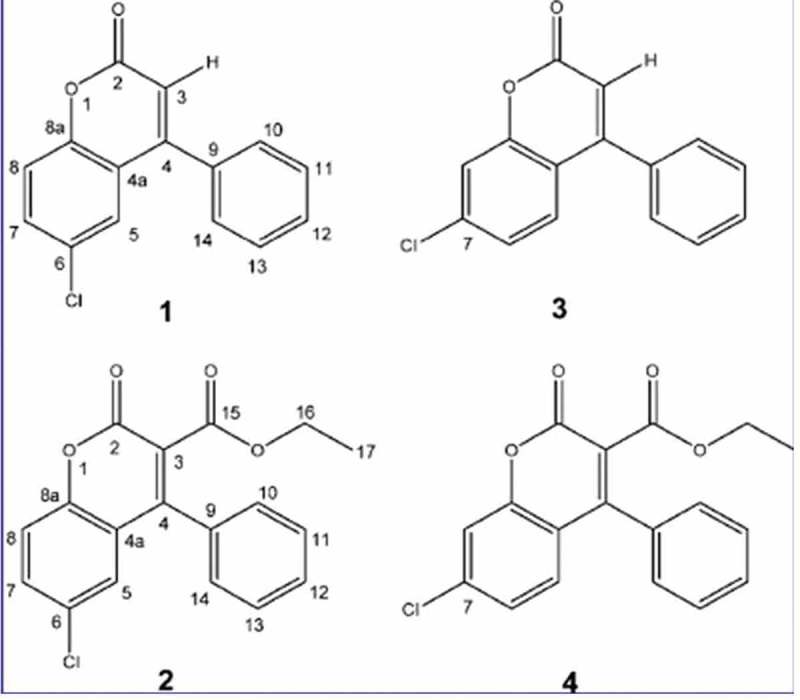
Naturally occurring coumarins (1–2) and newly synthesised analogues (3–4) from *F. officinalis*. (1) 6-chloro-4-phenyl-2*H*-chromen-2-one; (2) ethyl 6-chloro-2-oxo-4-phenyl-2*H*-chromen-3-carboxylate. From Hwang et al. (2013).

As it namely regards *F. officinalis*, (Hwang et al. 2013) tested both the two naturally occurring coumarins (showing Cl in position 6) and two other synthesised analogues (showing Cl in position 7) (Figure 2). The molecules were tested versus a broad panel of bacteria (*Staphylococcus aureus, Escherichia coli, Streptococcus pneumoniae, Enterococcus faecalis, Pseudomonas aeruginosa*), including several species in genus *Mycobacterium* (*M. smegmatis, M. chelonae, M. abscessus, M. marinum, M. kansasii, M. avium, M. bovis* and *M. tuberculosis*) as well as the yeast *Candida albicans*. Quaintly, only a narrow spectrum of significant antibacterial activity was observed, with lower MICs for *Mycobacterium tuberculosis*. Structural variations containing an ethyl ester reported higher anti-TB activity when Cl was in position 6 (naturally occurring) instead of 7 (synthesised). On the other hand, lowest MICs were observed in the newly synthetised compound 3 (Figure 1).

### 2.2. Lanostane-type triterpenoids (LTR)

**Figure 3.**
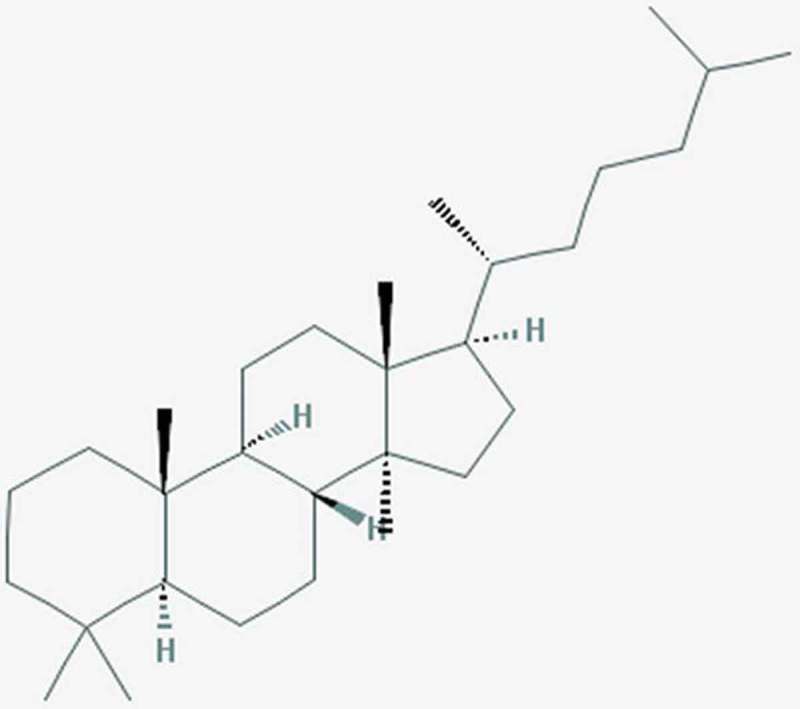
Lanostane or (5S,8R,9S,10R,13R,14S,17R)-4,4,10,13,14-pentamethyl-17-[(2R)-6-methylheptan-2-yl]-2,3,5,6,7,8,9,11,12, 15,16,17-dodecahydro-1H-cyclopenta[a]phenanthrene. From: https://pubchem.ncbi.nlm.nih.gov/compound/Lanostane.

**Figure 4.**
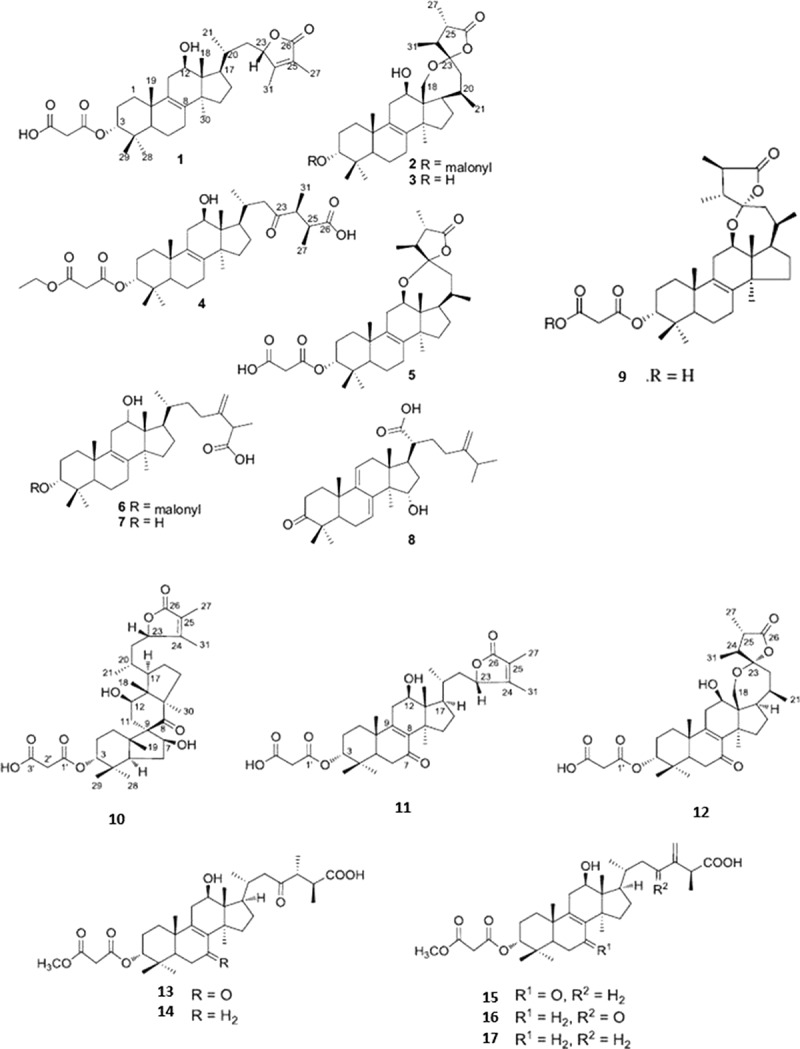
Lanostane-type triterpenoids isolated from *F. officinalis*. (1) 3-[2-(carboxyacetyl)oxy]-12-hydroxy24-methyl-lanost-8,24-dien-26,23-lactone named fomitopsin G; (2) 3-[2-(carboxyacetyl)oxy]-18,23-epoxy-12-hydroxy-24- methyl-lanost-8-en-26,23-lactone named fomitopsin H; (3) 18,23-epoxy-3,12,-dihydroxy-24-methyl-lanost-8-en-26,23-lactone named demalonyl fomitopsin H; (4) ethyl ester derivative of fomitopsin D; (5) fomitopsin F; (6) (25S)-(+)-12α-hydroxy-3αmalonyloxy-24-methyllanosta-8,24(31)-dien-26-oic acid; (7) fomeofficinic acid G; (8) 15α-hydroxy-3-oxo-24-methylenelanosta-7,9(11)-dien-21- oic; (9) fomitopsin C; (10) officimalonic acid A; (11) (3R, 12R, 23S)-3-carboxyacetyloxy-12-hydroxy-24-methyl-7-oxo-lanost-8,24-dien-26,23-lactone, named officimalonic acid B; (12) officimalonic acid C; (13) officimalonic acid D; (14) officimalonic acid E; (15) officimalonic acid F; (16) officimalonic acid G; (17) officimalonic acid H. Modified from Quang et al. (2005), Han et al. (2016) and Naranmandakh et al. (2018).

Lanostane-type triterpenoids (hereafter LTRs) formally derive from the cyclic terpenoid lanostane (Figure 3). They have been reported in several plant and fungal species including the basidiomes of *F. officinalis* (Kim et al. 2004; Wu et al. 2004; Feng et al. 2010; Han et al. 2016a; Isaka et al. 2017; Shi et al. 2017; Naranmandakh et al. 2018) (Figure 4). As particularly studied in genus *Fomitopsis*, some LTRs are also referred as fomitopsins (Table 2).

**Table 2.** Fomitopsins isolated from *Fomitopsis* species.

	F. feei	F. spraguei	F. officinalis
Fomitopsin A		Quang et al. 2005	Han et al. 2016
Fomitopsin B		Quang et al. 2005	
Fomitopsin C		Quang et al. 2005	Shi et al. 2017
Fomitopsin D	Isaka et al. 2017		
Fomitopsin E	Isaka et al. 2017		
Fomitopsin F	Isaka et al. 2017		Naranmandakh et al. 2018
Fomitopsin G			Naranmandakh et al. 2018
Fomitopsin H			Naranmandakh et al. 2018

Lanostane triterpenoids have been mostly investigated as it regards cytotoxicity and antitumor properties (Wu et al. 2013; Zhang et al. 2014), nevertheless antimicrobial activity has been reported too. Namely, fomitopsin D showed activity against herpes simplex virus type 1 (HSV-1) with an IC_50_ value of 17 μg/mL, whereas fomitopsins E and F resulted in inhibition of *Bacillus cereus* with MIC values of 6.25 μg/mL (Isaka et al. 2017). Moderate trypanocidal activity was reported versus *Trypanosoma congolense* by a panel of compounds including fomitopsin F (26 μM, IC_50_) and H (27.1 μM), a demalonyl derivative of fomitopsin H (12.5 μM), an ethyl ester derivative of fomitopsin D (15.0 μM) and especially the compound named 15α-hydroxy-3-oxo-24-methylenelanosta-7,9(11)-dien-21-oic acid (7 μM) (Naranmandakh et al. 2018). Trypanocidal activity by fomitopsin G was found negligible by the same authors. Antibacterial activity versus *S. aureus* has been reported also by lanostane derivatives in *F. rosea*, despite the same compounds have not been to date recognised in *F. officinalis* (Popova et al. 2009).

## 3. Antimicrobial properties of crude extracts

As the most bioactive metabolites in fungi show oxygenated groups, they can be easily recovered by ethanol extraction. Apart from pure hot water extraction, high graduation ethanol-water mixtures have been also the base of traditional medical use in several cultures, thus literature dealing with similar extraction methods provide a useful comparison term.

Hleba et al. (2016) tested 1-year-old crude ethanolic extracts from basidiomes of four medicinal species versus Gram-positive (*Bacillus thuringiensis, Staphylococcus aureus*) and Gram-negative (*Klebsiella pneumoniae, Enterobacter aerogenes*) bacteria by both MIC and disc diffusion methodology. Results showed that *F. officinalis* was the only species able to inhibit all the bacterial strains whereas MICs are very high. This is consistent with the previously mentioned (Hwang et al. 2013), yet target pathogens are different except for *S. aureus*.

Despite the attention paid by Russian and Ukrainian authors to *F. officinalis*, several full-texts are apparently not available to the international scientific community as unrecoverable on search engines. This problem has been partially overcome by the abundant reviewing production in the same Countries (Zaichenko et al. 2017). The work by Sidorenko and Buzoleva (2012) has its background in a patent for mycelia-based preparations against pseudotuberculosis thanks to the inhibitory effect towards *Yersinia pseudotuberculosis* (Sidorenko 2009a, 2009b). The same authors did not report instead any significant inhibition towards *E. coli, Pseudomonas putida, P. fluorescens, S. aureus, Listeria monocytogenes, Salmonella typhimurium*. Analogously, still unclear is the action of *F. officinalis* against the Gram-positive bacteria *Bacillus subtilis* and *B. anthracis*. Bacteriolytic effect towards *Vibrio* sp. has been related instead to the immunotropic activity shown by *F. officinalis* extracts, that is another topic producing an increasing literature (Yui et al. 2009; Kalinkevich et al. 2014; Vedenicheva et al. 2016).

By using an analogous methodological approach, Mithöfer and Boland (2012) is consistent with Coletto and Striano (2000), who observed inhibition of bacterial strains under examination, belonging to *B. cereus, B. subtilis, S. aureus, A. tumefaciens, E. coli, S. typhimurium*; weak inhibition of the yeast *C. albicans* was observed too.

Difference between efficiency of ethanolic and aqueous extracts was observed by Parkash and Sharma (2016) when testing *F. officinalis* against phytopathogenic microfungi (*Alternaria solani, Curvularia lunata, Aspergillus terreus* and *Fusarium oxysporum*) and bacteria (*E. coli* and *B. subtilis*). Pure ethanolic extract of *F. officinalis* was able to completely inhibit the growth of *A. solani* and *A. terreus*, whereas *C. lunata* and *F. oxysporum* were completely inhibited even when applying 1:4 diluted extract. Yet the same result was obtained when applying aqueous extract, *C. lunata* quaintly reported highest inhibition when applying 1:4 dilution. As it regards the antibacterial activity, highest inhibition was reached by applying the pure extract for *E. coli* and 1:4 dilution for *B. subtilis*. Aqueous extract only reported inhibition towards *E. coli* by 1:4 dilution. Despite basically consistent with literature on the existence of antimicrobial activity in *F. officinalis*, results by Parkash and Sharma (2016) are questionable both as concerning the methodology and significance of results themselves. As it regards the preparation of extracts authors in fact report the extremely low ratio of 0.01 g/10 mL between powdered basidiome and distilled water or ethanol (ethanol purity is not specified). Inhibition radius is investigated much below the millimetre after 3 days and the accuracy is therefore minimal.

A broad range of concrete applications relying on antimicrobial properties has been suggested by Stamets in distinct deposited patents exploiting the mycelial stage. Although including a great number of fungal species, the author always remarks the peculiarity of *F. officinalis*. In (Stamets 2011), dealing with “antiviral activity from medicinal mushrooms,” direct antiviral activity in HFF cells is reported by *F. officinalis* versus Cowpox and Vaccinia virus. Interestingly, the extract is reported to lack of agaric acid, whose bioactivity therefore remains unclear. In (Stamets 2014), dealing with “antiviral and antibacterial activity from medicinal mushrooms,” decrease in CFU is shown in *E. coli* and *S. aureus*. By relying on the antiviral activity of ethanol/water extracts from mycelia submitted to previous projects, Stamets (2018) deposited a patent concerning “antiviral activity of medicinal mushrooms and their active constituents.” Yet dealing with a plethora of species, the patent text remarks that 1–2% extract from *F. officinalis* is able to inhibit virus-induced cellular damage by 50% (EC_50_). 1:10^6^ diluted crude extract was reported as still effective against pathologically relevant virus of influenza A, influenza B and herpes. Selectivity index indicated extremely high activity. Significant inhibition is reported against *Mycobacterium tuberculosis* as well.

Further on, (Stamets 2016) deals with “integrative fungal solutions for protecting bees,” the target being the etiological agents of the colony collapse disorder (CCD). Yet showing not negligible activity in decreasing the total viral burden by 2 weeks, values of F. officinalis are low in comparison to several other polyporoid species under examination. More remarkable are the values reported as it regards the average percent improvement in longevity of bees even at 0.1% extract. As a whole, the LV Index (=the average percent improvement in bee longevity multiplied by the average percent decrease in total viral burden) indicates a competitive species for this purpose.

Antiviral activity against bird influenza (H5N1) and human influenza (H3N2) is also reported by Teplyakova et al. (2012) by applying *F. officinalis* aqueous extract (1 g basidiome biomass/5 mL water). Neutralisation index (ID_50_ ctrl-ID_50_ exp, logarithmic scale), respectively resulted 3.0 and 1.5, that is competitive yet not the highest in the test panel.

## 4. Conclusion

A rich literature consistently confirms the existence of antimicrobial properties by *F. officinalis*, namely as antiviral, antibacterial and trypanocidal. Such an activity has been specifically recognised in a few molecules, e.g. coumarines as antibacterial and lanostane triterpenoids as trypanocidal, although most metabolites have not been singularly tested yet. The different range of metabolites in basidiome and mycelium is of major concern in assessing the antimicrobial potential as well as to manage the possible cultivation or harvest.

The relative efficiency of *F. officinalis* in comparison to other medicinal species requires further investigation. Although few species can display contingently higher values in inhibition, *F. officinalis* may be more competitive when applied on very specific targets such as *M. tuberculosis* or *Y. pseudotuberculosis*.
